# Supplementary figures and images for: A telomere-to-telomere map of somatic mutation burden and functional impact in cancer
Source: bioRxiv. 2025 Oct 13:2025.10.10.681725. Preprint. [Version 1] doi: 10.1101/2025.10.10.681725 (PMC12632929; doi:10.1101/2025.10.10.681725)

Figure S1.

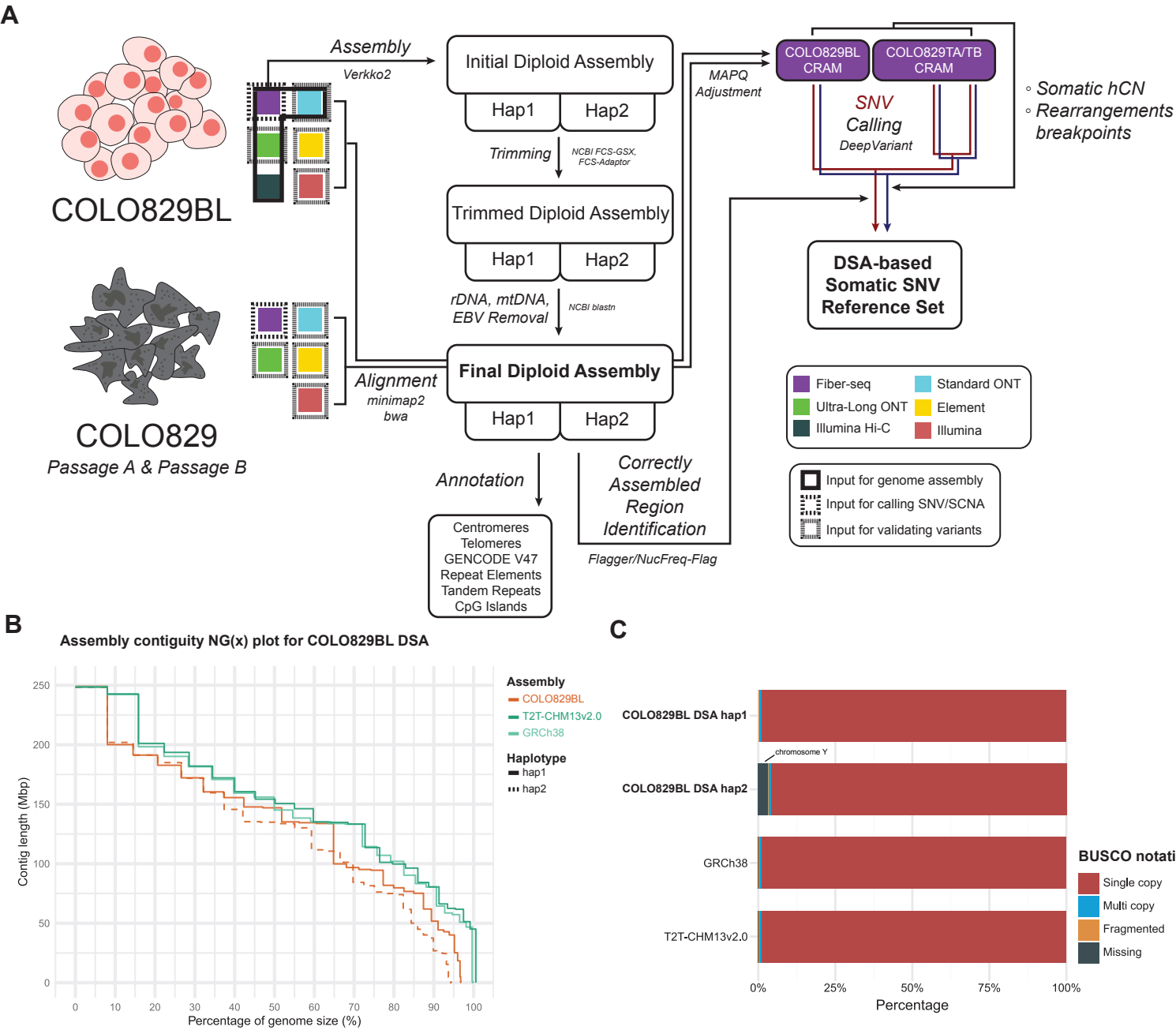

Supplement: Supplement 1 — Figure S1. Building near-T2T diploid DSA of COLO829BL and its quality metrics. A. Detailed diagram of constructing COLO829BL DSA and identifying somatic genomic events leveraging the assembly. B. Comparison of contiguity of the genome assembly using NG50 statistics. C. Quantitative measurement of assembly completeness across the DSA haplotypes, GRCh38 and T2T-CHM13. [file media-1.pdf]

**Figure S2.**

**A**

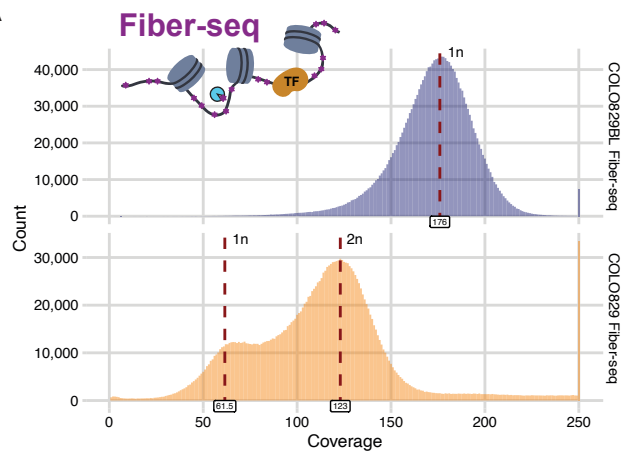

**B**

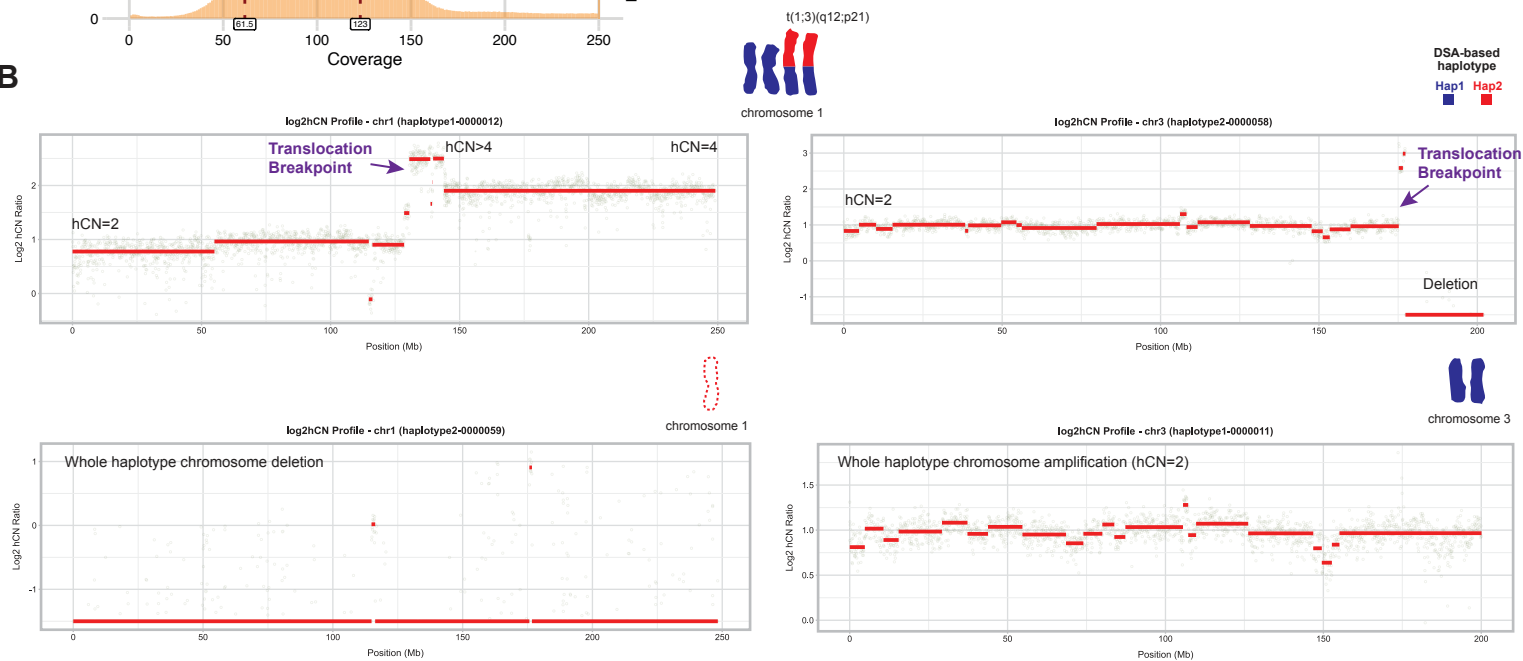

**C**

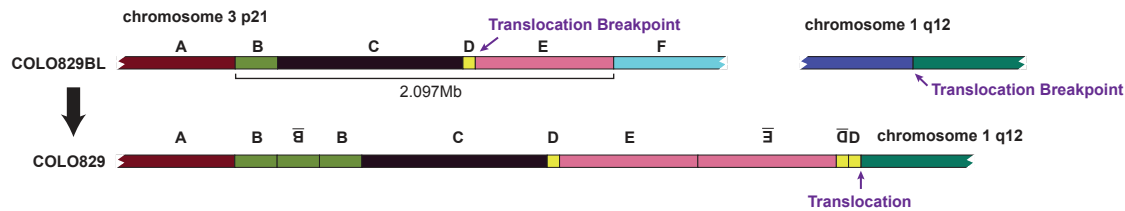

**D**

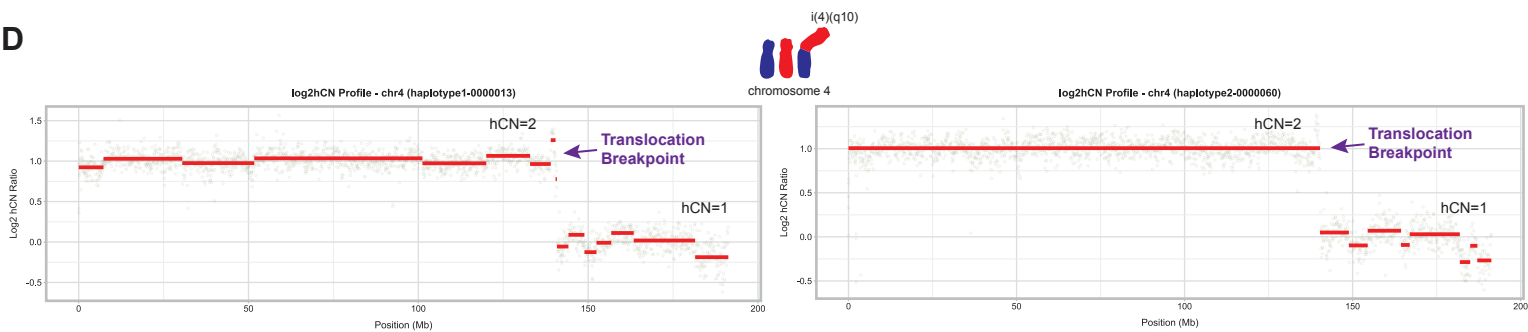

**E**

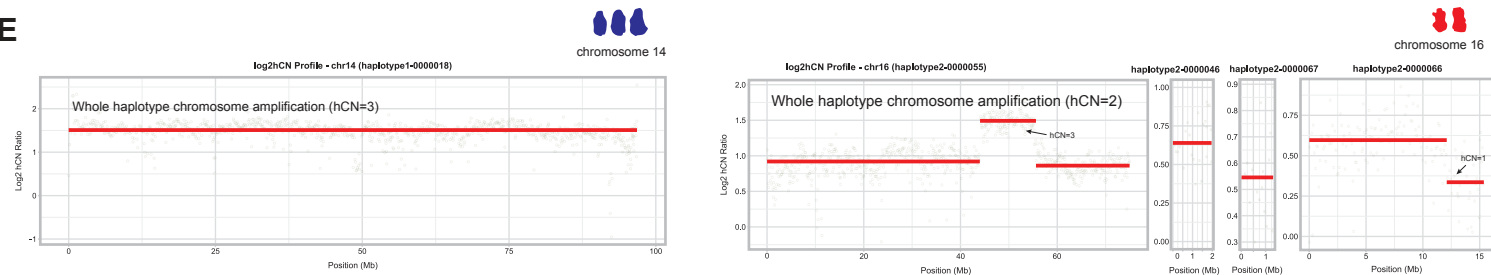

**F**

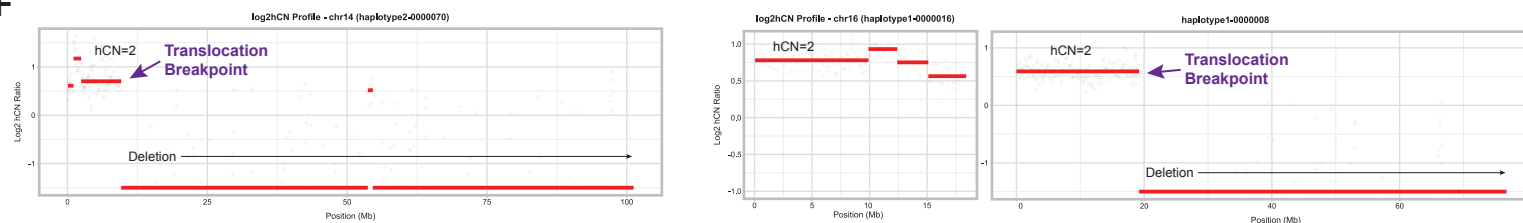

Supplement: Supplement 2 — Figure S2. Chromosomal rearrangements and haploid copy number alterations revealed by DSA-based analysis. A. Coverage histogram of COLO829BL and COLO829 (Passage B) Fiber-seq data across the diploid DSA. The coverage for 10 million bases was randomly drawn for each sample with sampling weighted by the length of the interval and excluding zero coverage regions. B. An example of identifying chromosomal-level rearrangements between chromosome 1 and chromosome 3 using log2hCN profiles across the DSA contigs. C. Schematic reconstruction of complex genomic rearrangements between chromosome 1 and 3 identified by the DSA-based analysis. D. Log2hCN profiles of q-arm of two chromosome 4 haplotypes forming isochromosome. E and F. Log2hCN profiles of chromosome 14 and 16, together with triplication and duplication events, respectively. The color of the ideograms on top of log2hCN ratio plot represents the haplotype of origin (haplotype 1:blue; haplotype 2:red). Each green dot represents a 100kb window “marker” with a red line indicating the median of each segment. [file media-2.pdf]

Figure S3.

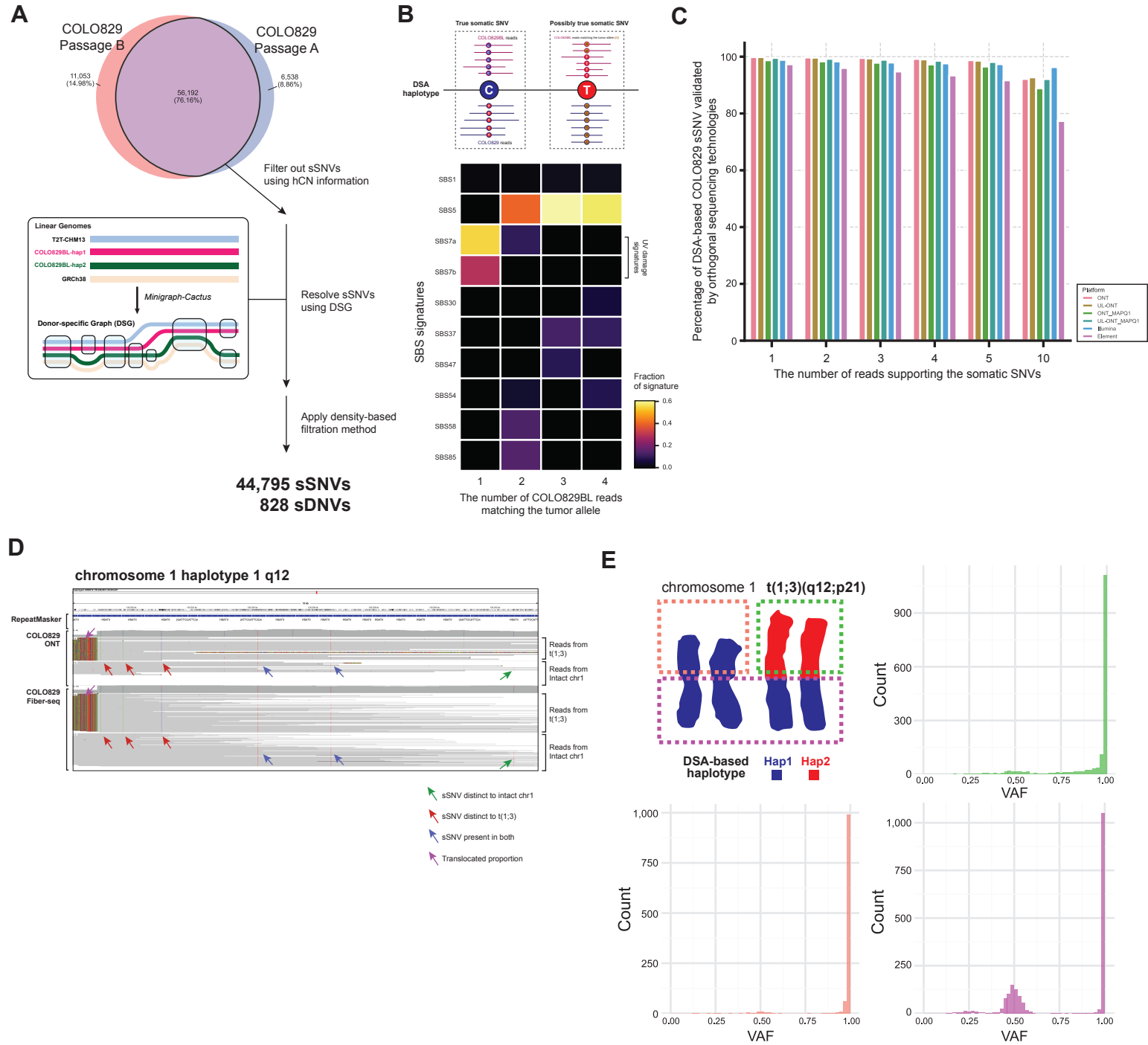

Supplement: Supplement 3 — Figure S3. Comprehensive somatic single nucleotide variant discovery and validation using the DSA. A. Sequential methods for identifying and refining sSNVs in COLO829 using the DSA. We integrated somatic haploid copy number data, a donor-specific graph (DSG), and density-based filtering to obtain a final set of 44,795 sSNVs and 828 sDNVs (see Methods). B. Heatmap showing the relationship between potential false-positive sSNVs and mutational signature composition. Each column represents the number of COLO829BL reads matching the tumor alleles, indicating likely false positives variants. Rows represent COSMIC single base substitution (SBS-96) mutational signatures (For this particular analysis, mutational spectrum normalization using 3-mer frequency (see Methods) was not applied and whole COSMIC SBS-96 signatures were used to reconstruct each mutational spectrum). C. Orthogonal validation rate of COLO829 sSNVs stratified by read support. D. IGV view of sSNVs present in chromosome 1 at the translocation breakpoints between chromosomes 1 and 3. Red arrows indicate sSNVs with ~0.5 VAF exclusive to the translocated fragment; green arrows mark sSNVs unique to the intact chromosome 1; blue arrows denote sSNVs present in both the intact chromosome 1 and the translocated segment. E. hVAF histogram of sSNVs stratified by involvement in translocation t(1;3). Lime green: sSNVs on chromosome 1 p-arm (not involved in translocation); salmon: sSNVs on chromosome 3 p-arm (involved in t(1;3) translocation); magenta: sSNVs on chromosome 1 q-arm (present on four copies, two of which are translocated to chromosome 3). [file media-3.pdf]

Figure S4.

A

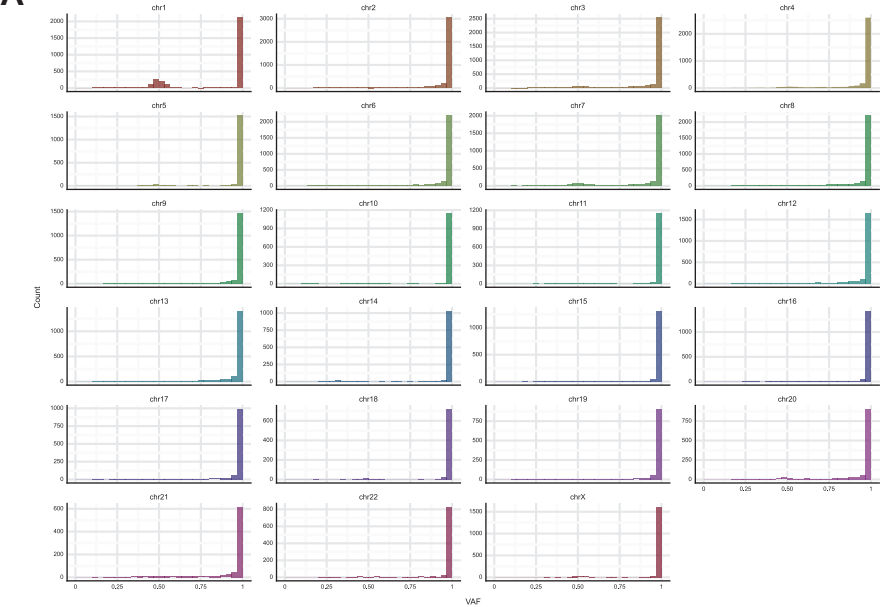

C

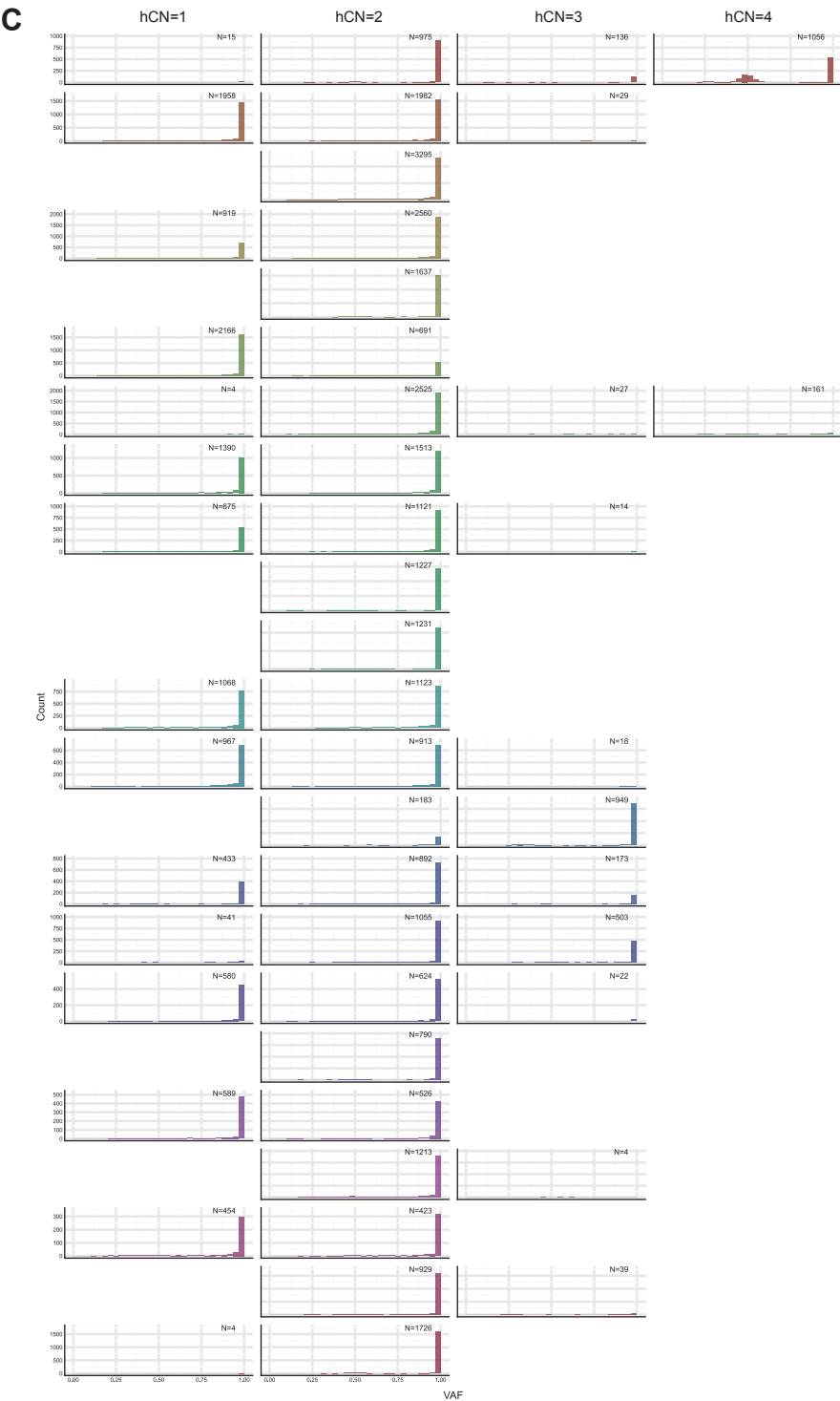

B

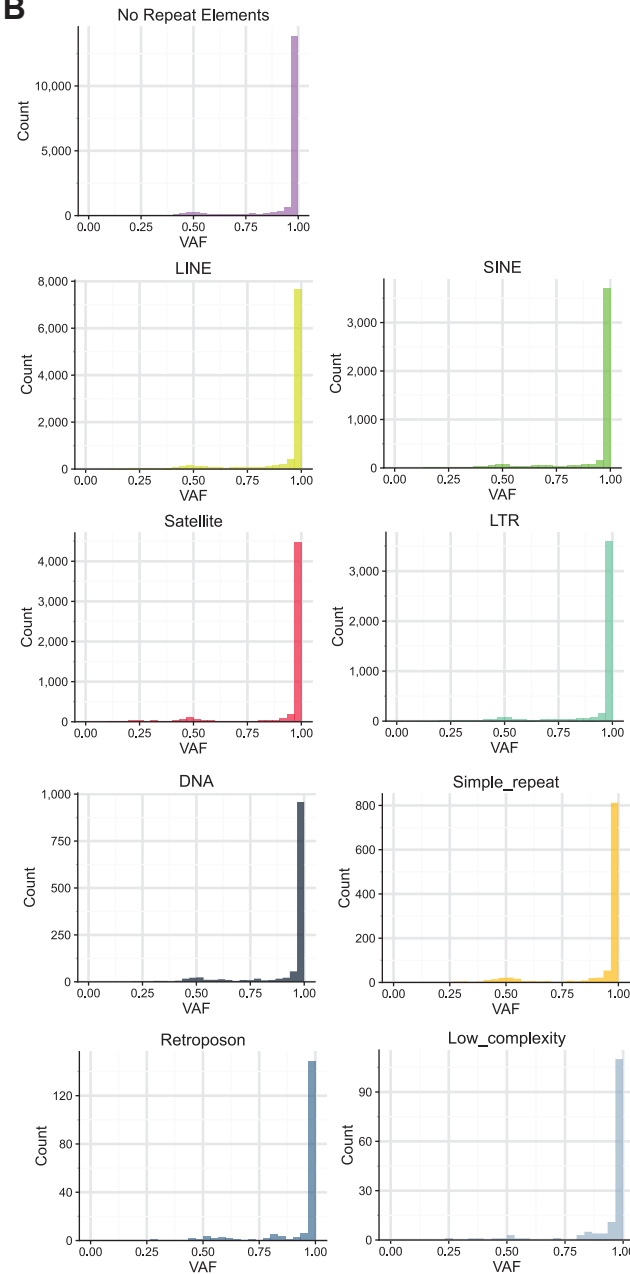

Supplement: Supplement 4 — Figure S4. Haploid variant allele fraction distribution pattern across genomic contexts. hVAF distribution of sSNVs across A. different chromosomal contigs in the COLO829BL DSA. B. different repeat classes and C. different chromosomal contigs separated by hCN states. [file media-4.pdf]

Figure S5.

A

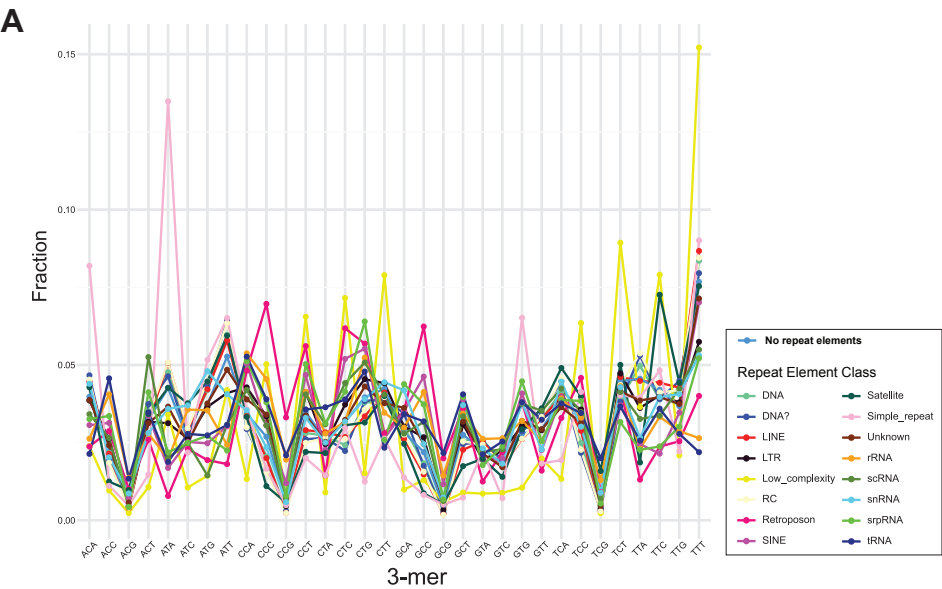

B

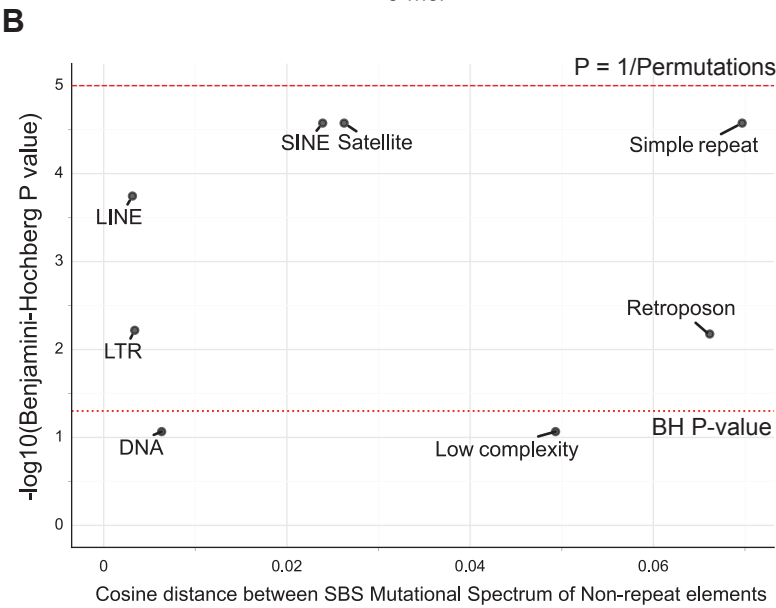

C

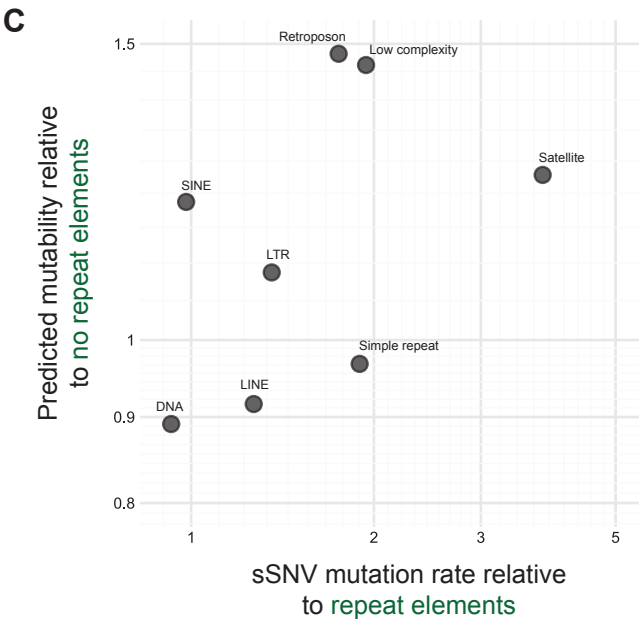

Supplement: Supplement 5 — Figure S5. Mutational spectrum analysis across various genomic contexts. A. 3-mer fraction across non-repetitive portions of the DSA and regions with different repeat elements. B. AMSD analysis results which compared mutational spectra between non-repetitive regions and different repetitive elements. C. Relationship between predicted mutability and observed sSNV mutation rate for relative to non-repeat elements across different repeat element classes. [file media-5.pdf]

Figure S6.

A

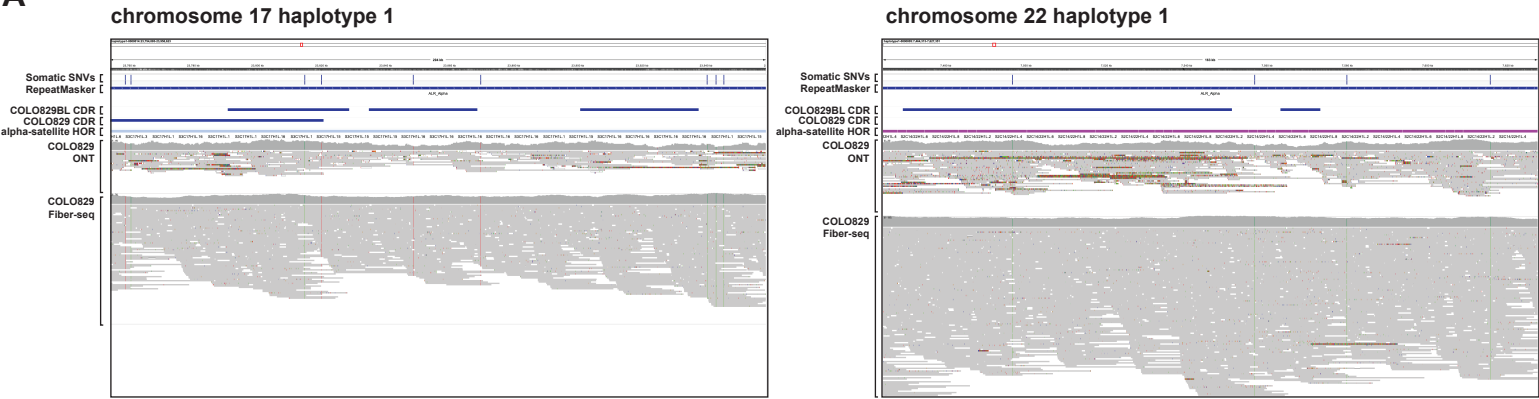

B

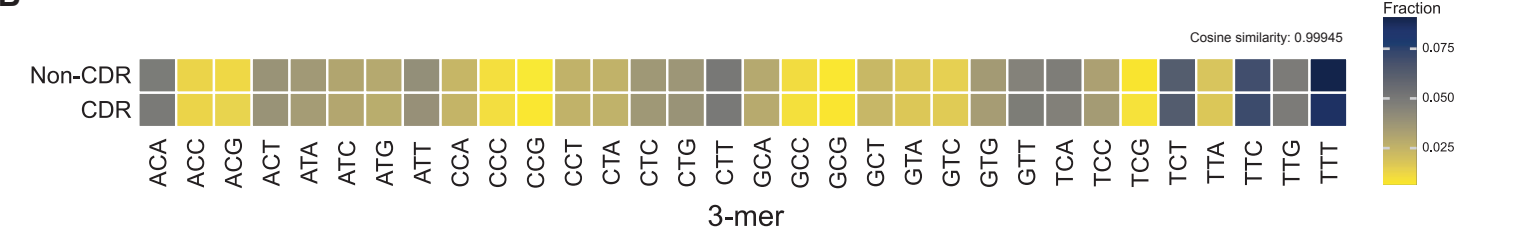

C

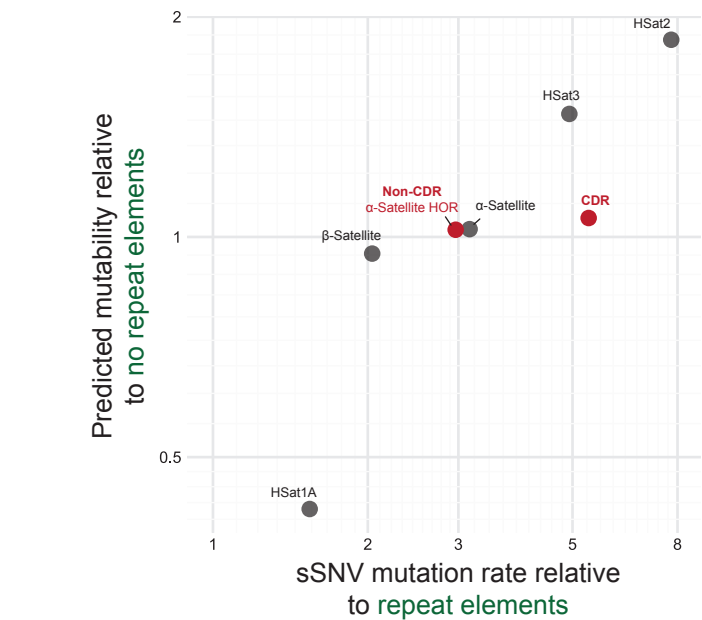

Supplement: Supplement 6 — Figure S6. Pattern of somatic SNVs in alpha-satellite regions of COLO829 centromeres A. IGV tracks showing sSNV distribution in CDR and non-CDR alpha-satellite regions for chromosomes 17 and 22. B. 3-mer fraction across CDR and Non-CDR alpha-satellite is largely identical with a cosine similarity of 0.99945. C. Predicted mutability vs. observed sSNV mutation rate for alpha-satellite repeats (CDR and non-CDR combined) relative to non-repeat elements. [file media-6.pdf]

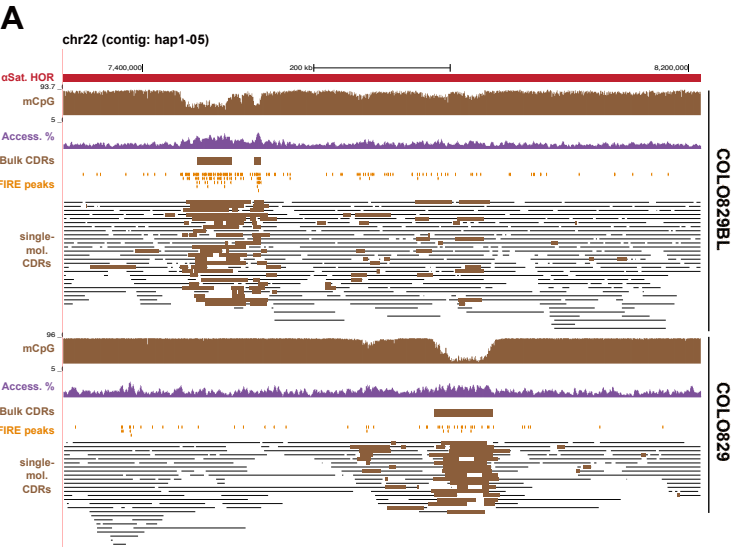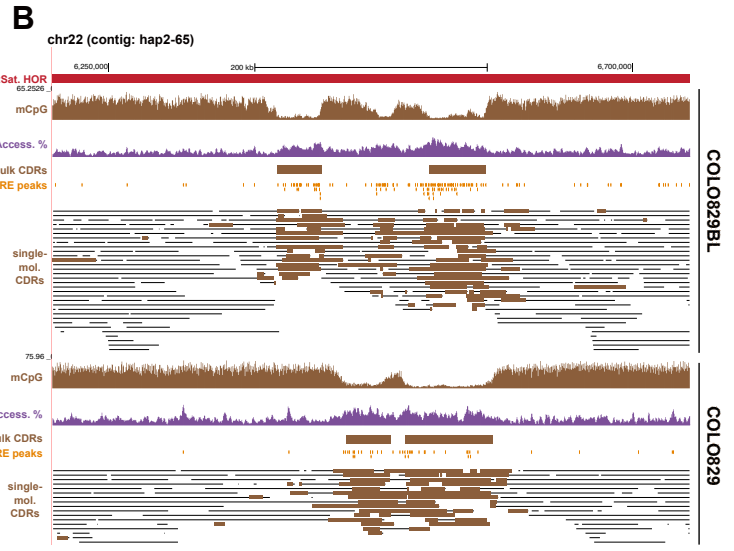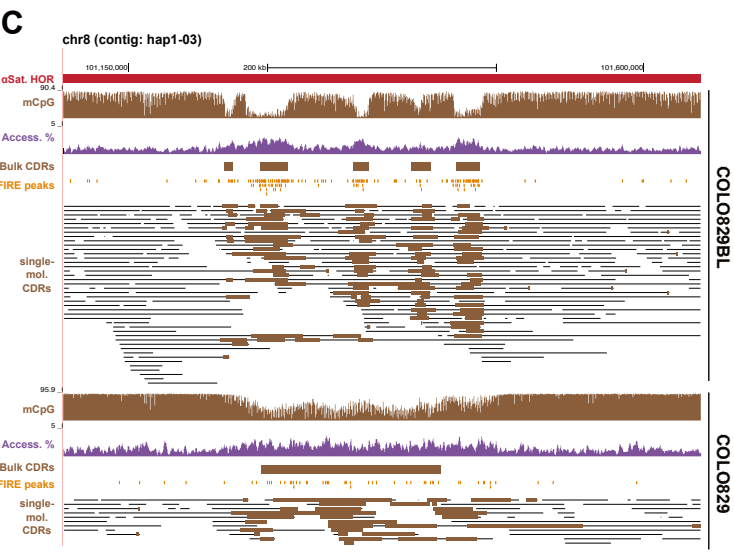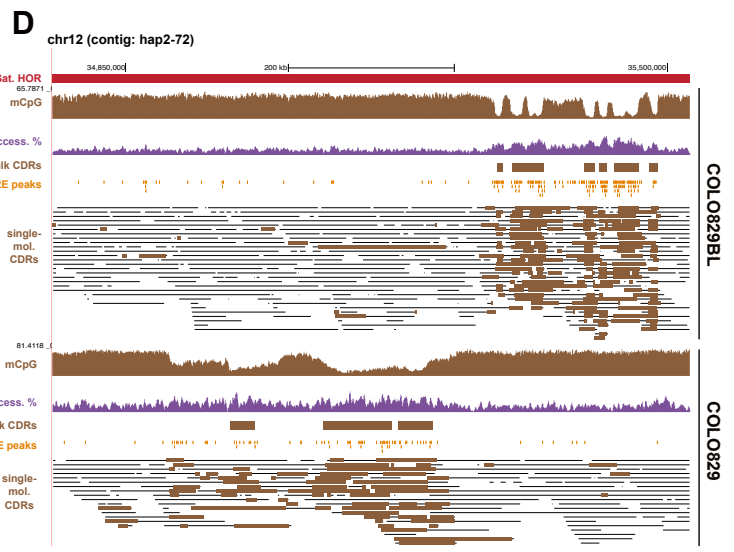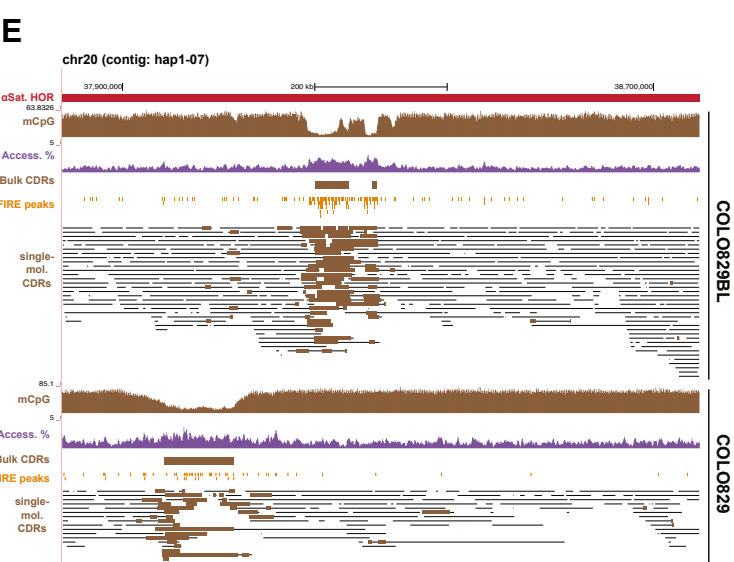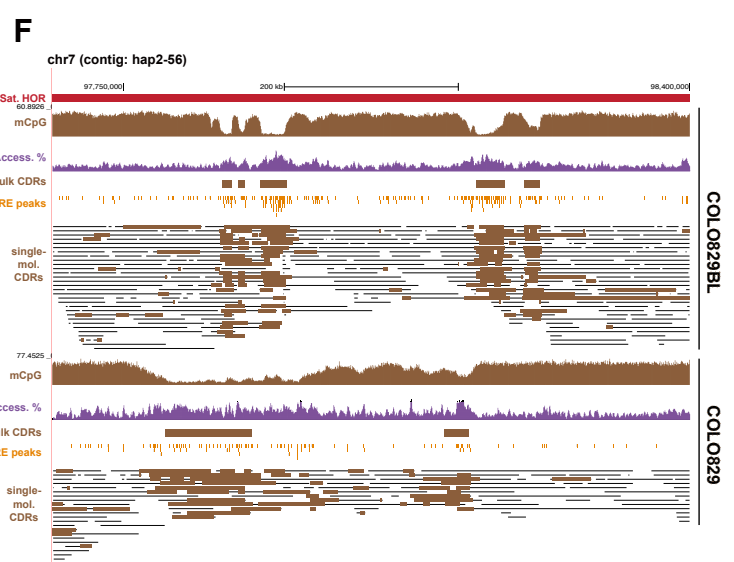

Supplement: Supplement 7 — Figure S7. Genome browser view of somatic rewiring of CDRs in COLO829. A-F Genomic loci displaying CDR restructuring. For each locus, in order from top to bottom, are alpha-satellite location, mCpG %, FIRE Accessibility %, Bulk CDR calls, FIRE peaks, and sing-molecule CDR calls. Top-most tracks represent COLO829BL, bottom tracks represent COLO829 (Passage B) [file media-7.pdf]

A

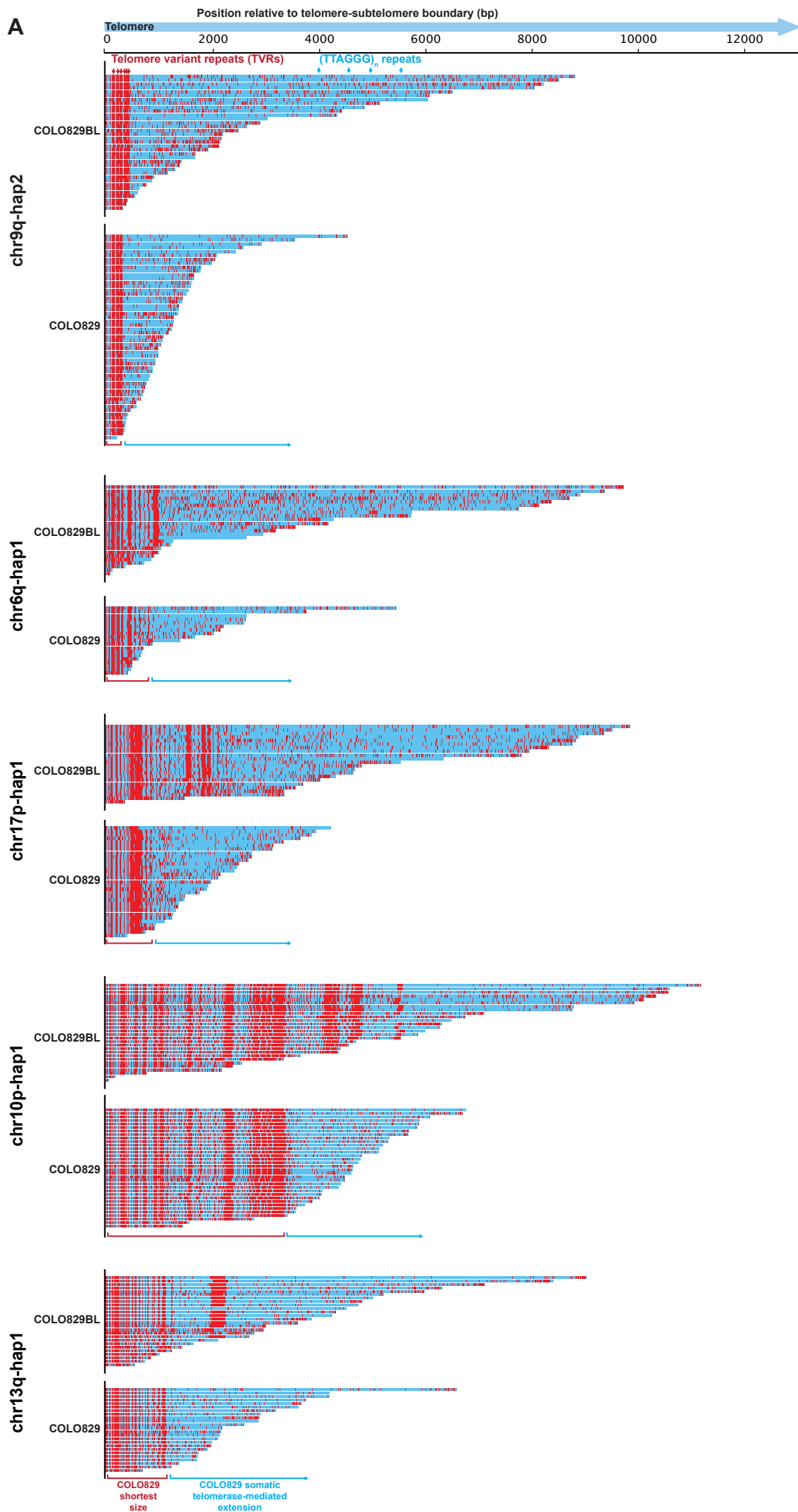

Supplement: Supplement 8 — Figure S8. Single-molecule-resolution architecture of telomeric restructuring events in COLO829. A. Restructured telomeric loci detected with PacBio Sequencing. Every individual bar represents a single-molecule sequencing with PacBio HiFi sequencing. Blue boxes represent canonical telomeric motifs (TTAGGGN), and red boxes indicate bases that cannot be classified within the canonical motif. The left most position for each individual read represents the boundary between sub-telomere and telomere, identified with seqtk telo. For every locus (left), COLO829BL is displayed on top, COLO829 displayed below. [file media-8.pdf]

**A**

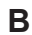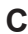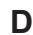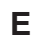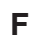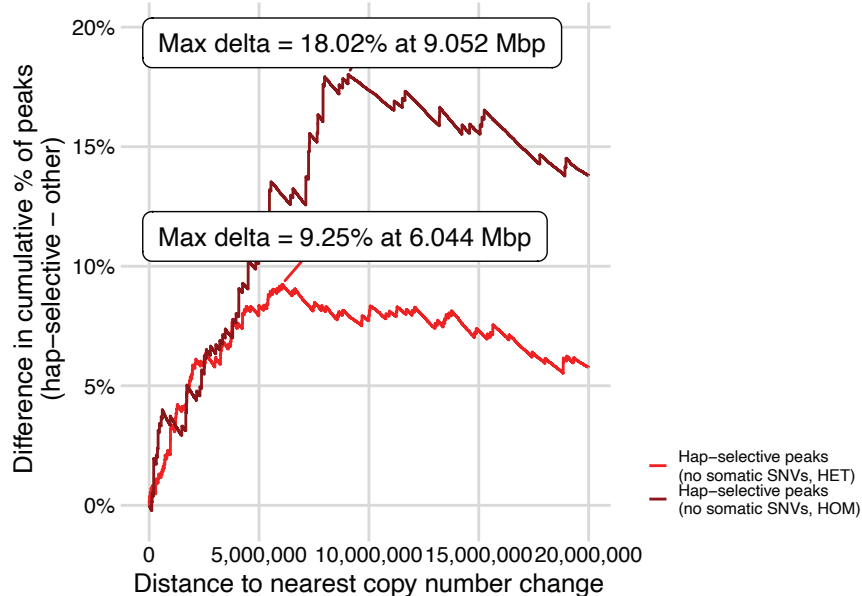

Supplement: Supplement 9 — Figure S9. Relationship between somatic alterations and epigenome rewiring. A. Identifying Fiber-seq-inferred regulatory elements (FIREs10) in the genome using Fiber-seq and fibertools. B. Associating FIREs in two corresponding haplotypes by leveraging the donor-specific assembly graph (DSG) constructed with T2T-CHM13, GRCh38 and COLO829BL diploid DSA. C. FIRE calls on individual reads at sites with haplotype-selective chromatin accessibility (HSCA) and a somatic variant. D. Difference in accessibility between the two haplotypes for sites with HCSA, both with and without intersecting somatic variants. E. Comparison of chromatin accessibility between haplotypes where one of the haplotypes has been amplified in COLO829 compared to the original COLO829BL copy number seen in the normal haplotype. F. The fraction of haplotype-selective Fiber-seq peaks without somatic variants or heterozygous germline variants (dark red) or other HSCA peaks (red) located within a specific distance (Mbp) from a change in copy number along the COLO829 genome and their difference from the cumulative distribution of all other FIRE peaks (see Figure 6D). [file media-9.pdf]
